# Superior Ophthalmic Vein Thrombosis Induced by Orbital Cellulitis: An Ophthalmic Emergency

**DOI:** 10.7759/cureus.19038

**Published:** 2021-10-25

**Authors:** Lu Chen, Uta S Guo, Gennadiy Grutman, Samy I. McFarlane, Parag Mehta

**Affiliations:** 1 Department of Cardiology, State University of New York Downstate Medical Center, Brooklyn, USA; 2 Department of Family Medicine and Community Health, Icahn School of Medicine at Mount Sinai, New York, USA; 3 Internal Medicine, New York-Presbyterian Brooklyn Methodist Hospital, Brooklyn, USA; 4 Department of Medicine and Endocrinology, State University of New York Downstate Medical Center, Brooklyn, USA

**Keywords:** bacterial infection, cavernous sinus thrombosis, ophthalmologic emergency, orbital cellulitis, superior ophthalmic vein thrombosis

## Abstract

Superior ophthalmic vein thrombosis (SOVT) is a rare ophthalmologic emergency. The most common etiologies include infection, trauma, inflammation, and malignancy, as well as thyroid-related orbitopathy. Early identification and timely intervention can lead to a significant decrease in complications that include cavernous sinus thrombosis (CST), vision loss, and death. This rare disease entity almost always makes its initial presentation to internal medicine or emergency medicine (EM) physicians. In this report, we present a case of SOVT that presented overnight to the emergency department for worsening right facial swelling and orbital pain. We discuss our experience with the evaluation and management of SOVT and provide a review of the currently available literature to emphasize the importance of obtaining a full history and physical examination, seeking early imaging studies, and ophthalmology consultation for patients with suspected SOVT.

## Introduction

Primary care and emergency medicine (EM) physicians serve as the initial medical contact for patients with a broad spectrum of diseases. Superior ophthalmic vein thrombosis (SOVT) is a rare ophthalmologic emergency almost always first presented in the primary care or emergency setting. The presentation of SOVT varies depending on the underlying pathology, with the most common etiologies being infection, trauma, neoplasm, and vasculitis [1]. The most commonly reported cases of SOVT are associated with infectious etiology of surrounding structures, such as orbital cellulitis (OC) [2-8], sinusitis [9,10], dental abscess [7,11], and cutaneous infection [12-14]. Due to the strong association with orbital cellulitis, suspicion of SOVT is high if the physical examination is suggestive of optic nerve or orbital involvement [1]. Reported complications include permanent impairment or loss of vision [5,7,8,15] and death [4,11,14]. 

We describe a case of OC-induced SOVT initially treated as conjunctivitis and, after further investigation, successfully managed with intravenous (IV) antibiotics and anticoagulation. The purpose of this case report is to emphasize the importance of obtaining a full history with a focused physical examination, seeking early imaging studies, and ophthalmology consultation for patients with suspected SOVT.

## Case presentation

A 44-year-old male with uncontrolled diabetes mellitus (DM) type II secondary to medication noncompliance presented overnight to the emergency department for worsening right hemifacial swelling and sharp orbital pain with flu-like symptoms that started 10 days prior. He reported right-sided ocular pain but denied changes in visual acuity or color perception. Physical examination revealed periorbital swelling, tenderness, and erythema that extended down to the right maxilla. Phlebitis was appreciable along the bilateral frontal sinuses and extended inferiorly to the nasal ridge. The ocular examination revealed chemosis, ptosis, and conjunctival injection in the right eye. The patient was admitted to medical service and started on acyclovir and clindamycin for suspected herpes zoster-induced conjunctivitis with possible sinusitis.

Despite aggressive management, reoccurring febrile episodes were noted overnight, and cellulitis appeared to worsen the following morning. Further history revealed that the patient works in construction and frequently wipes his face with gloved hands. He also reported medication noncompliance due to a lack of insurance. A thorough examination revealed ocular pain and pain with extraocular movement, with no diplopia. Visual acuity, color perception, and pupillary light reflex were bilaterally intact. Laboratory studies were remarkable for leukocytosis of 14,600/mL with neutrophil predominance and glycohemoglobin of 11.1%. The remaining laboratory studies, including renal function and hepatic function, were unremarkable.

**Figure 1:**
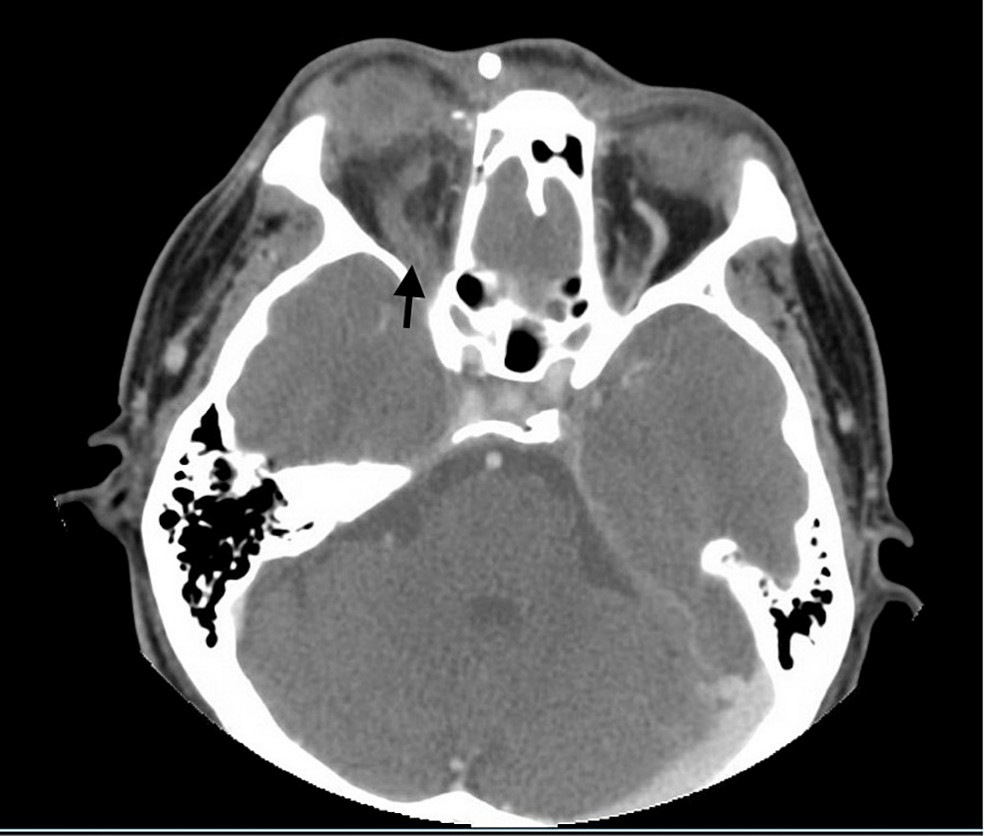
Right-sided superior ophthalmic vein thrombosis. Enlargement of the superior ophthalmic vein and lack of contract uptake (arrow).

**Figure 2:**
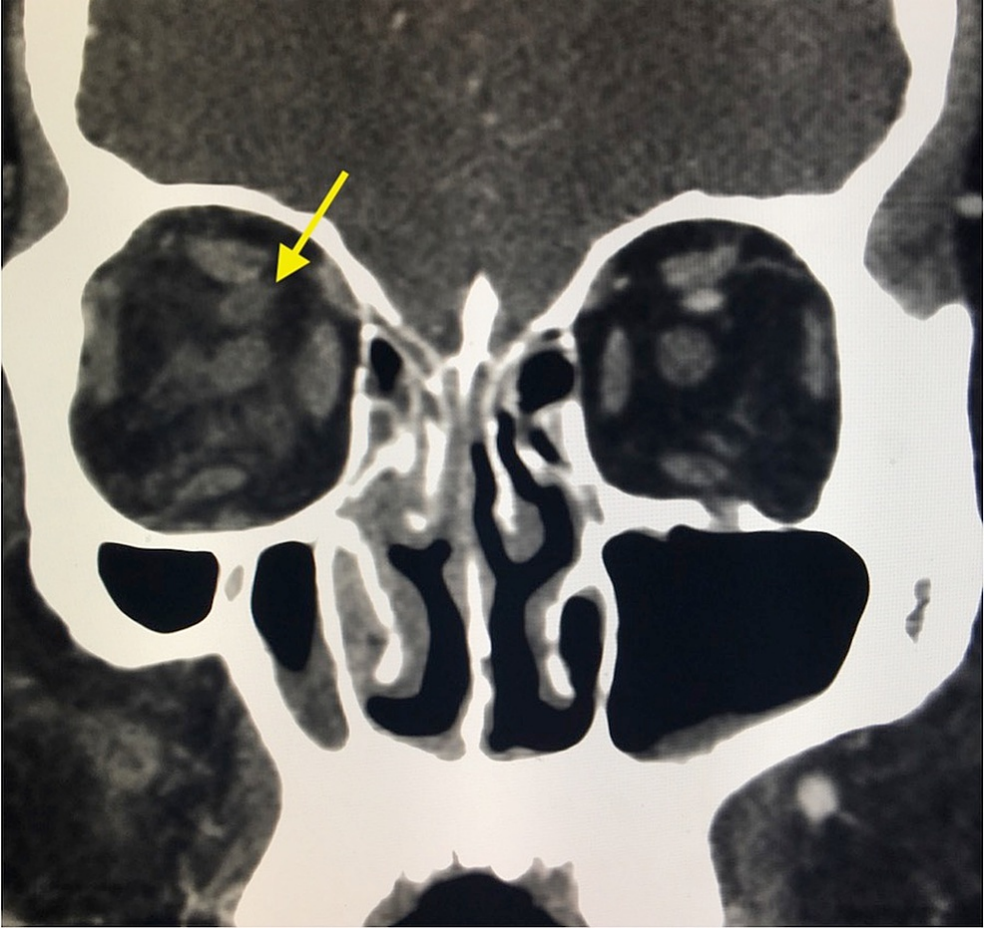
Coronal view of the right-sided superior ophthalmic vein thrombosis (arrow), with fat stranding suggestive of congestive edema and inflammation.

Maxillofacial computed tomography (CT) with IV contrast was obtained due to the concern of orbital involvement (Figures 1 and 2). CT scan demonstrated proptosis of the right eye, thrombosis of the right superior ophthalmic vein, and possible cavernous sinus thrombosis (CST). Orbital cellulitis was suspected, and anticoagulation with unfractionated heparin was initiated. Ophthalmology consultation was requested, and the patient was transferred to the intensive care unit in a tertiary care center. The patient was managed conservatively and discharged home on oral antibiotics with outpatient follow-up. At three-month follow-up, the patient reported complete resolution of symptoms with no impairment of visual acuity. He also reported medication compliance at this time.

## Discussion

SOVT is a rare but serious clinical condition that requires immediate intervention. Delay in care can result in permanent blindness and fatality. Infection-induced SOVT usually presents with orbital cellulitis, frequently as a result of direct inoculation from sinusitis [1]. Due to the valveless nature of the superior ophthalmic veins and facial veins, a seemingly innocuous superficial infection in the danger triangle can spread posteriorly to the cavernous sinus leading to a CST or meningitis [16]. In our patient with a relative immunocompromised state from uncontrolled DM, frequent face wiping at the construction site became the likely nidus of infection.

The distinguishing feature of SOVT is optic nerve or orbital involvement. The impairment of visual acuity, color perception, and pupillary light reflex can suggest optic neuropathy. In addition, pain or paralysis with eye movements, proptosis, or diplopia suggests inflammation of extraocular muscles and thus orbital involvement [17,18]. In the absence of symptoms of optic neuropathy and orbital cellulitis, superficial infections such as preseptal cellulitis are more likely. Based on available literature detailed in Table 1, SOVT can present in a wide heterogeneity in age, sex, comorbidities, and inciting infection. However, every patient diagnosed with infectious SOVT had more than one symptom or sign of optic nerve or orbital involvement (Table 1). The majority of patients present with unilateral ocular complaints, although bilateral ocular involvement has been reported as well [19]. Suspicion of either optic nerve or orbital involvement should prompt immediate imaging studies and urgent ophthalmology consultation to rule out SOVT and CST [18].

**Table 1:** Review of available publications on superior ophthalmic vein thrombosis, including presentation, inciting bacteria, antibiotics used, and clinical outcome.

Author and year published	Patient age, gender, and inciting bacteria	Presenting symptoms and physical examination	Bacteria identified	Antibiotic and other anti-infectious agents	Diagnostic modality	Anticoagulation	Steroid (dose)	Outcome
Our paper	44, M, orbital cellulitis	Ocular pain, periorbital swelling, tenderness, chemosis, ptosis, conjunctival injection	Not mentioned	Acyclovir and clindamycin	CT showed SOVT and possible CST	Heparin	Not used	Patient reports complete resolution of symptoms at three-month follow-up
Syed et al. (2016) [14]	25, F, left facial cellulitis secondary to methamphetamine injection into left cheek	Obtunded, L>R chemosis, anisocoria	Methicillin-resistant *Staphylococcus aureus* (MRSA)	Broad spectrum, refined to vancomycin and rifampin	CT angiography found multiple acute infarcts, MRI confirmed multiple venous thrombosis	Heparin	Not mentioned	Patient suffered multiple embolic CVA and cavernous sinus thrombosis, death
Rohana et al. (2009) [13]	16, F, nasal furunculosis	Severely infected eyes, chemosis, mucoid discharge, restricted eye motion, no afferent pupillary defect, visual acuity intact	Staphylococcus aureus	Cloxacillin initially, ceftazidime for two weeks	CT orbit with and without showing SOVT and CST	Heparin to warfarin	Not mentioned	Complete recovery
Akiyama et al. (2013) [8]	65, M, decayed tooth, right-sided facial cellulitis, orbital cellulitis	Eye pain, eyelid edema, right-sided visual loss, without severe limitation of eye movement	Not mentioned	Meropenem, clindamycin	CT showed SOVT, MRI showed no intracranial involvement	Heparin	Betamethasone	Repeat imaging showed bilateral SOV within normal range, patient reports right-sided vision loss
Aggarwal et al. (2017) [11]	40, F, tooth extraction by an unregistered practitioner	Right facial swelling diplopia, dilated pupil, proptosis, periorbital ecchymosis, chemosis, loss of vision, abducens nerve palsy	Not mentioned	Imipenem, clindamycin, ceftriaxone, chloramphenicol, metronidazole	CT showed SOVT and CST	Not used	Dexamethasone (8 mg every eight hours)	Patient underwent oral and maxillofacial surgery but succumbed to the complications of the disease, death
Embong et al. (2006) [7]	43, M, dental infection, left orbital cellulitis, right parapharyngeal abscess	Decreased visual acuity, afferent pupillary light defect, swelling of eyelids, proptosis, ophthalmoplegia, increased intraocular pressure	Mixed growth	Ceftazidime, vancomycin, metronidazole, topical ciprofloxacin	MRI showed CST and SOVT	Heparin to warfarin	Not mentioned	Patient underwent emergency parapharyngeal drainage and received intravenous antibiotics, patient showed symptom improvement
Pendharkar et al. (2011) [20] (N = 2) Case 2: Aseptic etiology	Case 1: 64, F, dental infection	Chemosis, proptosis, blindness, ophthalmoplegia, dilated/sluggish pupil	Staphylococcus aureus	Antibiotics	MRI showed CST and SOVT	Not mentioned	Not mentioned	Death
Ghosheh et al. (2006) [6]	47, M, orbital cellulitis, MRSA, endocarditis	Decreased vision, ophthalmoplegia, chemosis, proptosis, no afferent pupillary defect	MRSA	Vancomycin, piperacillin/tazobactam	CT inconclusive, MRI showed SOVT	Not mentioned	Not mentioned	After treatment, patient had improvement in extraocular movements, proptosis, and chemosis
Ogul et al. (2014) [10]	20, M, ethmoidal sinusitis	Periorbital swelling, bilateral proptosis, extraocular movement restricted, visual acuity intact, no afferent pupillary defect	Not mentioned	Antibiotics for 14 days	MRIs showed SOVT with inferior ophthalmic venous thrombosis	Heparin and subsequent oral anticoagulation	Not mentioned	Patient completed antibiotic course and showed symptom improvement
Parmar et al. (2009) [5]	14, M, orbital cellulitis from ethmoid sinusitis	Headache, photophobia, stiff neck, proptosis, complete CN6 palsy, partial CN3 palsy	Not mentioned	Vancomycin, metronidazole, piperacillin/tazobactam	CT showed SOVT and CST, CT venogram and MRI confirmed CT findings	Heparin to lovenox	Not mentioned	Patient’s mental status and ophthalmic finding improved
De Lott et al. (2013) [21]	59, M, multiple abscesses in the left masticator space involving the left masseter and medial pterygoids	Reduced vision of the right eye, double vision on left gaze, no afferent pupillary defect, right-sided ptosis, proptosis, limited right eye motion, right intraocular pressure elevated than the left eye	Streptococcus anginosus	Vancomycin, clindamycin, levofloxacin, changed to ceftriaxone and metronidazole upon speciation	MRI showed SOVT and CST	Heparin and discontinued after six weeks	Not mentioned	Patient’s visual acuity and ocular motility returned to normal
Gomi et al. (2005) [9]	58, F, left sphenoid sinusitis leading to SOVT on the right side	Severe right eye pain, periorbital swelling and erythema, right eye chemosis, proptosis, ptosis, visual acuity affected, extraocular movement limited by pain	Methicillin-sensitive *Staphylococcus aureus* (MSSA)	Piperacillin/tazobactam then amoxicillin/sulbactam as outpatient; vancomycin, ceftriaxone, and metronidazole upon admission; oxacillin replaced vancomycin upon speciation	CT showed right-sided SOVT and left sphenoid sinusitis with no CST, MRI confirmed CT findings	None	Dexamethasone	Patient underwent left sphenoid sinus drainage and received six weeks of antibiotic therapy. At follow-up, patient’s right eye symptoms resolved
Walker et al. (2002) [4]	80, F, ethmoid sinusitis	Proptosis, diplopia, restricted and painful eye motion, mild chemosis, swollen, tender, red eyelid	MSSA	Cipro with acyclovir, ceftriaxone with flucloxacillin, gentamicin added later	CT orbit and head showed SOVT and no CST, MRI confirmed CT findings	Enoxaparin	Not mentioned	Patient died 14 days after admission from massive hemorrhage
Sanchez et al. (1997) [12] (N = 2)	Case 1: 41, F, nasal vestibulitis with facial, orbital cellulitis	Chemosis, proptosis, decreased visual acuity	Not mentioned	Cefalotin, then changed to clindamycin	CT showed right CST and bilateral superior ophthalmic vein thrombophlebitis	Not mentioned	Dexamethasone	Patient showed complete resolution of all visual changes after three weeks of IV antibiotics
	Case 2: 52, F, furunculosis of the nose	Eyelid edema, severe chemosis, pain, difficulty opening eyes, CN6 nerve paralysis	Staphylococcus aureus	Cefalotin, then changed to clindamycin	CT showed left SOVT with no CST	Not mentioned	Not used due to patient history of DM and HTN	Patient showed full recovery after seven days of hospital stay
Cumurcu et al. (2013) [3]	65, F, ethmoidal sinus, orbital cellulitis	Blurred vision, proptosis, chemosis	Blood and conjunctival culture negative	Ceftazidime, amikacin, metronidazole	CT showed SOVT with no CST, MRI and cerebral angiography confirmed findings	Heparin	Not mentioned	Patient showed improved visual acuity, proptosis, and chemosis with normal eye movements after 14 days of treatment
Mandić et al. (2017) [15] (N = 2) Case 2: Aseptic etiology	Case 1: 61, F, orbital cellulitis, underwent functional endoscopic sinus surgery	Sluggish pupil, orbital congestion, periorbital edema, chemosis, vision loss, increased intraocular pressure	Coagulase-negative *Staphylococcus*, *Serratia marcescens* (hospital acquired)	Amoxicillin and clavulanic acid, then ceftriaxone	CT showed a high suspicion of septic SOVT, confirmed with CT angiography	Yes, no specific drug specified	Yes, no specific drug specified	Patient underwent emergency sinus decompression; upon discharge, patient had normal periocular status, but no light perception on affected eye

In patients with infectious SOVT, the most common bacteria was *Staphylococcus aureus* (seven out of 10 cases) [4,6,9,12-14,20], including two cases of methicillin-resistant *Staphylococcus aureus* (MRSA) [6,14]. Coagulation-negative *Staphylococcus* and *Streptococcus anginosus* were also reported [15,21]. Therefore, the initial choice of antibiotic should be broad spectrum, with coverage for *Staphylococcus aureus*. Coverage for MRSA should be individualized; previously reported cases of MRSA infection were both associated with the intravenous use of illicit drugs [6,14].

Initial anticoagulation and steroid use have been reported, although the benefit of either therapy is unclear at this time [1]. Anticoagulation therapy was used in 11 cases and considered in one additional case. The initial agents of anticoagulation used were heparin or low-molecular-weight heparin. Steroid therapy, most commonly dexamethasone, was used in five cases. The paucity of SOVT cases makes it difficult to standardize the use of anticoagulation of steroid therapy. The decision of either therapy should be individualized to a specific patient.

## Conclusions

SOVT is a rare medical emergency often initially presented in the primary care or emergency care setting. A focused history and physical examination to rule out orbital involvement and optic neuropathy are critical. Early detection and treatment of SOVT prevent progression to CST and its detrimental sequelae. Fortunately, the majority of the cases reported complete recovery. Suspected cases of SOVT should trigger early imaging studies and urgent ophthalmology consultation to minimize detrimental complications.
